# The Contribution of In Vivo Mammalian Studies to the Knowledge of Adverse Effects of Radiofrequency Radiation on Human Health

**DOI:** 10.3390/ijerph16183379

**Published:** 2019-09-12

**Authors:** Andrea Vornoli, Laura Falcioni, Daniele Mandrioli, Luciano Bua, Fiorella Belpoggi

**Affiliations:** Cesare Maltoni Cancer Research Center, Ramazzini Institute, Castello di Bentivoglio, via Saliceto 3, Bentivoglio, 40010 Bologna, Italy; falcionil@ramazzini.it (L.F.); mandriolid@ramazzini.it (D.M.); bual@ramazzini.it (L.B.); belpoggif@ramazzini.it (F.B.)

**Keywords:** radiofrequency radiation, in vivo experimental studies, carcinogenicity, reproductive/developmental toxicity

## Abstract

The proliferation of cellular antennas and other radiofrequency radiation (RFR) generating devices of the last decades has led to more and more concerns about the potential health effects from RFR exposure. Since the 2011 classification as a possible carcinogen by the International Agency for Research on Cancer (IARC), more experimental studies have been published that support a causal association between RFR exposure and health hazards. As regard cancer risk, two long-term experimental studies have been recently published by the US National Toxicology Program (NTP) and the Italian Ramazzini Institute (RI). Despite important experimental differences, both studies found statistically significant increases in the development of the same type of very rare glial malignant tumors. In addition to carcinogenicity, reproductive organs might be particularly exposed, as well as sensitive to RFR. In this work, we reviewed the currently available evidence from in vivo studies on carcinogenicity and reproductive toxicity studies in order to summarize the contribution of experimental research to the prevention of the adverse effects of RFR on human health.

## 1. Introduction

Since mobile phone usage has become an integral part of everyday life for the vast majority of the population, unprecedented human exposure to radiofrequency radiation (RFR) from conception until death has been occurring in the last two decades. Consequently, there is an increasing public interest in the possible health risks derived from mobile phone use and base station-related exposure.

RFR, which includes radio waves and microwaves, correspond to 30 kHz–300 GHz the electromagnetic spectrum. RFR has enough energy to move atoms in a molecule around or cause them to vibrate, but not enough to ionize (to detach electrons from atoms or molecules), which is, therefore, known as non-ionizing radiation. The important properties of non-ionizing radiation include the frequency at which it is generated, measured in megahertz (MHz) or gigahertz (GHz), and the intensity of the waves, or the specific absorption rate (SAR), which is the rate of energy absorption per unit mass of biological tissue [1]. RFR can cause tissue heating when having sufficient intensity, which is the principle of the microwave oven functioning. Given the ability of RFR to heat tissues, the toxic effects of RFR are often pointed to thermal effects only.

Among the effects induced by RFR, tissue heating is a well-established and biologically plausible mechanism: When the RFR exposure occur at levels high enough, the absorption of energy by a biological system could overcome its capability to regulate the body temperature. Assuming the phones are not emitting more than permitted, typical human exposures to RFR occur at intensities that are not capable of inducing significant tissue heating if devices are used according to the manufacturers’ recommendations for use.

Those biological changes occurring when body temperature increase is below 1 °C are referred to as nonthermal RFR effects. Temperature variations within the range of 1 °C are considered as thermal noise [2]. There is an ongoing debate regarding whether nonthermal biological effects can occur as a result of exposures to low-intensity RFR. Even though some authors have suggested that exposure to low-intensity RFR would not be able to induce significant biological effects through a plausible non-thermal mechanism [3,4,5], numerous are the studies that associate specific biological effects to RFR exposures at levels considered below those expected to result in a measurable amount of tissue heating. The mechanisms of interaction between living organisms and RFR have not yet been well characterized, but several mechanisms have been proposed, besides tissue heating; among these are included the induction of ferromagnetic resonance, the alteration of ligand binding to hydrophobic sites in receptor proteins, and above all the most plausible is the forced-oscillation of free ions in all biological cells resulting in irregular gating of voltage-gated ion channels in cell membranes [6]. Moreover, exposure to low levels of RFR can cause small temperature changes in localized areas of exposed tissues leading to conformational changes in temperature-sensitive proteins and inducing the expression of heat-shock or stress-response proteins [2].

In 2011 the International Agency for Research on Cancer (IARC), part of the WHO, declared RFR to be “possibly carcinogenic to humans”, group 2B [2,7], basing primarily on limited evidence in humans that long-term users of mobile phones held to the head resulted in an elevated risk of developing brain cancer, and limited evidence from animal studies that RFR exposure lead to cancer.

One of the biggest concerns is that RFR might have reproductive and developmental adverse effects, in particular, disturbing testicular function and altering sperm parameters. It is indeed probable that the penetration of RFR into the testis may be more pronounced than other tissues, given the lower protection of this organ by tissue, in comparison to others. It is well known that the temperature of the testicles is 2 °C to 3 °C lower than the rectal one, and the right temperature for spermatogenesis is considered to be 35 °C [8]. The habit of keeping the cellphone in the trouser pocket or the prolonged use thereof may have an impact in generating hyperthermia of the scrotum, as well as oxidative stress, which represent the main damage generation mechanisms [9], besides non-thermal effects.

This review aims to address the current knowledge of both the carcinogenic and the reproductive/developmental hazards of RFR emerged from in vivo experimental studies. Firstly, cancer bioassays have been reviewed. Based on the animal model, reviewed articles were separated by paragraphs in studies on rats, mice and other models (including trans-genic models). Those experimental studies in which wild type or transgenic/tumor-prone strains of rats and mice were subjected to RFR long-term exposure of at least one year were taken into consideration. Those experiments with an exposure duration beneath the 12 months were deliberately excluded, since they can not be considered reliable carcinogenicity studies [10]. 

Secondly, apical endpoints investigated in reproductive and developmental toxicity peer-reviewed studies were used for PubMed selection of relevant in vivo animal studies. Hence, those experiments in which the authors evaluated the effect of RFR exposure towards reproductive system health were reviewed. Based on the animal model, reviewed articles were separated by paragraphs in studies on rats, mice and other mammalian models.

## 2. Cancer-Related In Vivo Investigations

Since billions of people are exposed to the potential carcinogenic risks of RFR, studies in laboratory animals must be as sensitive as possible for really being informative. The Organization for Economic Co-operation and Development (OECD) and the NTP have drawn up specific guidelines for the conduction of carcinogenicity studies [11,12]. Among the specifications for design and conduct of experimental studies to evaluate carcinogenic potential of xenobiotics, such as the physical agent RFR, are in example the following: (1) Each dose group and concurrent control group should contain at least 50 animals of each sex, (2) at least three dose levels should be used (in addition to the concurrent control group), and (3) the period of dosing and duration of the study should be of at least 24 months [10].

### 2.1. Rats

Among rat studies, La Regina et al. (2003), using a carousel system, tube-exposed Fischer 344 (F344) rats for 4 h/day, 5 days/week, for 24 months. Two different types of RFR (835.62 MHz FDMA, 847.74 MHz CDMA) at one brain SAR level of 1.3 ± 0.5 W/kg each were applied to the animals. Each group (2 RFR and 1 sham) consisted of 160 rats (80/80). No significant differences between treated and sham-exposed animals were found in the incidence of any spontaneous tumors [13].

Anderson et al. in 2004 exposed F344 rats to 1.6 GHz RFR. Animals were divided into three groups of treatment: One group was sham exposed, and two groups were subjected to a far-field RFR Iridium signal. Exposures started prenatally at levels resulting in fetal brain SAR of 0.16 W/kg. After parturition, 90 restrained animals per sex and per group underwent 2 h/day, head-first, near-field exposures for 5 days/week until the rats were two years old, with calculated levels of brain SAR corresponding to 0.16 W/kg and 1.6 W/kg and near-field sham controls. It was not observed any statistically significant difference among the three experimental groups as for the incidence of neoplastic lesions [14]. 

In 2007, Smith et al. divided 1170 Han Wistar rats among 65 male and 65 female per group, exposing the animals for 2 h/day, 5 days/week for up to 24 months at three nominal SARs of 0.44, 1.33, and 4.0 W/kg to Global System for Mobile Communications (GSM) or Digital Cellular System (DCS) wireless communication signals. No adverse reaction was observed following exposure to different levels of both the signals. Particularly, except for those results that the authors reputed as isolated, trivial observations not related to the treatment, this study did not report any statistically significant finding in the incidence, multiplicity, latency, or type of any primary cancers that can be attributable to RFR neither in male nor in female rats [15]. 

Also, five two-years promotional cancer studies involved promotion of N-Ethylnitrosourea (ENU)-induced cancer, four in F344 rats and one in SD rats [16,17,18,19,20]. RFR carrier frequencies ranged from 836 to 1950 MHz with different modulations. Nevertheless, none showed an increase nor in ENU-initiated brain cancer promotion nor any other statistically significant observation. It should be noted that in all the five studies, a carousel system to restrain rats was used, and this likely have presented a stress factor, complicating the interpretation of the results.

Among the studies conducted in Sprague-Dawley (SD) rats, in 1992 Chou et al. exposed 200 SD rats for 25 months, 21.5 h per day, to pulse modulated 2450-MHz RFR at 0.144–0.4 W/kg of whole body SARs. Exposed animals showed a statistically significant increase in the incidence of primary malignant tumors, compared to the same number (*n* = 200) of sham-exposed rats. Among those neoplasms found in the exposed rats were thyroid cancer and malignant lymphoma. The importance of these results is given by the fact that the thyroid gland is one of the most RFR-exposed organs during mobile phone usage, especially during a call [21]. 

The study by the US National Toxicology Program (NTP) was the largest rodent bioassay carried out by this U.S. government Institution. SD rats were exposed to RFR in special chambers for up to two years, with exposure began in the womb. The RFR exposure was intermittent, 10 min on and 10 min off, for a total of about 9 h a day, with exposure levels of 1.5, 3, and 6 W/Kg/bw. Rats were exposed at a frequency of 1900 MHz to total body RFR from two technologies, CDMA and GSM [22]. 

The Ramazzini Institute (RI) study was the largest long-term bioassay ever performed exploring the health effects of RFR, comprising 2448 rats. The whole-body exposure for 19 h/day of male and female SD rats to a 1.8 GHz GSM far field of 0, 5, 25, 50 V/m, started prenatally and lasted until natural death [23].

NTP doses were established to mimic the localized exposure on body tissues from a cell phone placed near the body—and were, therefore, particularly higher than those used by the RI, which were, instead, similar to those found in our living and working environment to mimic the full-body human exposure generated by mobile telephony base antennas. Despite these differences, recently, both the studies found statistically significant increases in the development of the same type of very rare glial malignant tumors [22,23].

During the use of cordless and handheld mobile phones, the brain is the main target of RFR. An increase in gliomas of the brain, the same tumor found in people after long-term cell phone use, was observed in both NTP and RI studies, although a statistically significant increase was observed only by NTP. The results published by the RI highlighted a statistically significant increased incidence of a very rare glial tumor of the heart, the Schwannoma, in male rats treated at the highest dose (50 V/m). This is the same type of tumors found to be increased by NTP using far higher exposure levels, tumor involving the same hystotype of the acustic nerve (vestibular) neurinoma observed in humans after intensive mobile phone use in epidemiological studies [2]. Both in the NTP and in the RI studies, the increase in the risk of Schwannomas was low. 

In the NTP study, an increased number of male rats bearing adrenal gland tumors was also considered to be related to exposure. The RI publication only documents brain and heart findings. Data from the other organs are about to be published.

The studies discussed in this section are summarized in Table 1.

### 2.2. Mice

In addition to the study conducted in SD rats, NTP also conducted a carcinogenesis study in B6C3F1/N mice. Animals were located in special chambers and treated with RFR for up to two years, with exposure began at 5–6 weeks of age. The RFR exposure was intermittent, 10 min on and 10 min off, for a total of about 9 h a day, with exposure levels of 2.5, 5 m and 10 W/Kg. The whole body of the mice were exposed to RFR at a frequency of 1900 MHz, from two technologies, CDMA and GSM. In male or female mice, exposure to cell phone RFR did not significantly increase the incidence of any neoplastic lesions [24].

Using B6C3F1 mice, Tillmann et al. (2007) experimentally evaluated the possible carcinogenic effects of RFR at 902 and 1747 MHz, respectively of the GSM and of the DCS standards. Mice were restrained and exposed over a period of 2 years for 2 h/day, 5 days/week to three different whole-body averaged SAR levels of 0.4, 1.3, 4.0 W/Kg body weight, or were sham exposed. No statistically significant increase in the incidence of any particular tumor type was observed in the RF exposed groups as compared to the sham exposed group [25]. 

RFR cocarcinogenic effect was explored in the 2010 tumor promotion study by the same authors. Mice were exposed for two years to Universal Mobile Telecommunications System (UMTS) fields at intensities of 0 (sham), 4.8, and 48 W/m^2^. The low-dose group (4.8 W/m^2^) was subjected to additional prenatal ethylnitrosourea (ENU) treatment of 40 mg/kg body weight, and showed an increase in the rate of lung tumor and in the incidence of lung carcinomas in comparison with the control group treated only with ENU [26].

Similar results were obtained from a follow-up study in which mice were treated with RFR at SAR levels of 0 (sham), 0.04, 0.4, and 2 W/kg [27]. The incidence of lung and liver tumors and of malignant lymphomas was significantly higher in RFR exposed animals, compared to control (sham-exposed) rats.

The studies discussed in this section are summarized in Table 2.

### 2.3. Other Models

Among the studies conducted on transgenic/tumor-prone animal strains, in 1997, Repacholi et al. observed an increase in β-cell lymphoma in transgenic mice exposed to pulsed digital RFR. Such increase was observed for female pim1 (carrying a lymphomagenic oncogene) mice after 18 months of exposure to a modulated 900 MHz GSM signal of two 30-min periods per day, at SAR exposure levels ranging from 0.008 to 4.2 W/kg, averaging 0.13–1.4 W/kg [28]. This result was not replicated by the studies of Utteridge et al. (2002) and Oberto et al. (2007) that had similar designs [29,30]. 

In particular, in 2002 Utteridge and collaborators used a modulated 898.4 MHz GSM signal for exposing female heterozygous pim1 transgenic (lymphoma-prone) mice (*n* = 120 per group) in a “ferris-wheel” system. Tube-restrained animals were RFR-radiated for 1 h/day and 5 days/week within two years at 0, 0.25, 1.0, 2.0, and 4.0 W/kg as whole body SAR levels. In addition, the study included a non-restrained cage control group, and a positive control, treated only with 50 mg/kg ENU. All in all, contrary to what observed by Repacholi, no lymphoma increase was observed in the RFR exposed groups [29].

In the study by Oberto et al. (2007) that the authors defined as “an extension of a previously published study conducted by Repacholi et al.”, female Pim1 transgenic mice (*n* = 50/group/sex) were tube-restrained and treated for 1 h/day, 7 days/week, up to 18 months, at whole body SAR levels of 0, 0.5, 1.4, 4.0 W/kg. The results of this bioassay do not suggest any effect due to pulsed 900 MHz RFR exposure on the onset of tumors, thus, disproving once again, the results by Repacholi et al., under the conditions used [30].

The possible co-carcinogenic effect of 2450 MHz RFR exposure and 3, 4-benzopyrene was explored by Szmigielski et al. in 1982 in two different types of transgenic mice. Balb/c and C3H/HeA mice were exposed to RFR from 6 weeks of age for 2 h a day, 6 days a week, up to 1 year of age, either before (over 1 or 3 months) or concurrently to the treatment with benzopyrene (over five months). RFR at the frequency of 2450 MHz showed to induce cancer promotion at both 50 and 150 W/m^2^. The findings of this study revealed an increase in chemically-induced and spontaneous tumors [31].

The studies discussed in this section are summarized in Table 3.

## 3. Reproductive/Developmental Toxicity In Vivo Investigations

Both OECD and NTP recently updated their study guidelines for reproductive and developmental toxicity adding various functional endpoints for assessing how an agent can affect the reproductive and endocrine status of animals [32,33]. Here, we used those endpoints for articles selection by Pubmed to assess the state of art of the literature about RFR toxicity potential, distinguishing for studies on the male and female reproductive system, and other reproductive/developmental endpoints, discriminating by strains of experimental animals. In particular, the apical endpoints used for literature search on Pubmed were the following: Assessment of sperm quality, viable litter size/live birth index, neonatal growth, neonatal survival indices, prenatal mortality, weight and morphology of reproductive organs, estrous ciclicity, precoital interval, mating and fertility indices and reproductive outcome, duration of gestation, parturition, landmarks of sexual maturity (vaginal opening, urogenital distance, balano-preputial separation), functional toxicities and CNS maturation, qualitative and quantitative physiologic endpoints revealing unique toxicities of pregnancy and lactation, nesting and nursing behavior, sexual behavior, sex ratio in progeny, oocyte quantification.

### 3.1. Male Reproductive System

#### 3.1.1. Rats

One of the first studies investigating cellphone RFR was conducted in SD rats and examined just the effects, due to exposure on testicular and sperm function. Rats were restrained in cages built ad hoc in Plexiglas, and mobile phones were placed 0.5 cm under the cages. Cellular phones with frequencies between 890 and 915 MHz were activated at a SAR level of 0.52 W/kg for 20 min/day for up to one month. No statistically significant difference between treated and control animals was reported in this study for any of the analyzed parameters [34]. 

Testicular histological changes in rats exposed to 848.5 MHz RFR for 12 weeks were explored by Lee et al. (2010). Male SD rats underwent two daily exposure periods of 45-min, with an interval period of 15 min. The authors investigated the concentrations of MDA in the testis and epididymis, the sperm count in the cauda epididymis, the frequency of the stages of spermatogenesis, the germ cell count, and the appearance of apoptotic cells in the testis. According to the results of this study, rat spermatogenesis was not influenced by any detectable adverse effect [35].

Two years later, the same researchers examined the effects of combined exposure to 848.5 MHz CDMA and 1950 MHz WCDMA RFR on most of the same parameters analyzed in their previous study. SD rats underwent RFR exposure for 45 min a day, 5 days a week for a total of 3 months, at an average whole-body SAR of 4.0 W/kg for both the frequencies. Based on the findings, they concluded that not even such simultaneous exposure resulted in any observable adverse effect on rat testicular function [36].

In 2011, Imai et al. performed a study on adolescent SD rats, exposing the animals to 1.95 GHz RFR at a whole-body SAR level of 0.4 W/kg for 5 h/day, 7 days/week, for a total of 5 weeks. The exposure period corresponded with the reproductive maturation of the rats. No difference in weights of the epididymis, seminal vesicles, testis or prostate was observed between exposed and control rats. Sperm count in the epididymis and testis was not influenced by the treatment, and no alterations in the sperm motility, morphology, or in the histological appearance of seminiferous tubules, including the spermatogenic cycle stage, was observed between treated and untreated animals [37].

Among “non-influential” studies conducted in Wistar rats, in 2007 Ribeiro et al. investigated the effects of subchronic RFR exposure emitted by GSM cellular phone (1.835–1.850 GHz) for 1 h/day for 11 weeks on the testicular function. Epididymal sperm count, epididymal and testicular weight, total testosterone in the serum and lipid peroxidation levels in these organs, such as various qualitative testicular histopathological end points were analyzed. No statistically significant difference was found between treated and control animals for all the considered endpoints [38].

A study by Trošić et al. was aimed to establish the possible negative impact of RFR exposure on Wistar rat male reproductive health. The research group evaluated the count, motility and form of spermatozoa from the cauda epididymis, as well as the histology of the testis. Animals were total body irradiated for 1 h/day over two weeks to 915 MHz RFR, at the average SAR value of 0.6 W/kg. An haemocytometer was used for microscopically determining the quality, quantity and structure of free sperm cells taken from the epididymis. This study revealed no statistically significant changes in any of the evaluated endpoints [39].

In 2007, the study by Yan et al. reported a statistically significant decreased motility of the epididymal sperm of SD rats treated with 1.9 GHz RFR, in comparison to controls. Furthermore, treated rats, unlike the unexposed, showed abnormal clumping of spermatozoa. Compared to the previous study by Dasdag et al., the apparent opposition in the results obtained by Yan et al. may be likely due to the longer treatment period to which rats from the same strain were subjected in this experiment. In fact, the experimental rats, restrained in special plastic holding tubes, were daily exposed to cell phone RFR for three hours, followed by 30 min of non-exposure outside the tubes, and, to follow, by another exposure period of 3 h more [40].

In 2014, Qin et al. used adult male SD rats to explore the circadian effects of the exposure to RFR on reproductive functional markers. Animals in circadian rhythm (based on melatonin measurements) underwent RFR exposure at 1.8 GHz, at a SAR level of 0.0405 W/kg, for 2 hours a day, for 32 days in total. Circadian rhythms were found disrupted in animals exposed to RFR, as well as testosterone levels, daily sperm production and sperm motility were found decreased, the activity of γ-GT and ACP down-regulated, and the mRNA expression of cytochrome P450 and steroidogenic acute regulatory protein were altered in comparison to sham exposed rats. These results show that RFR exposure can negatively impact male reproductive functional markers, both in terms of total daily levels and in terms of circadian rhythmicity [41].

Finally, in 2019, Guo et al. explored the effects of 30 days exposure to pulsed modulated RFR at 220 MHz on the sperm quality in male adult SD rats. Calculated average whole body and testis SAR values were 0.030 W/kg and 0.014 W/kg, respectively. Compared to controls, the sperm quality in the treated group decreased significantly. Sperm cells quality was assessed by measuring the survival rate, the number and the abnormalities of spermatozoa. After the treatment, the level of Leydig cells secreting factor assessed by ELISA decreased significantly, whereas the Western blotting-assessed levels of caspase 3, cleaved caspase 3, and the BAX/BCL2 ratio in the testis markedly increased. Moreover, the levels of secreted factors of Sertoli cells assessed by ELISA and the testis morphology by HE staining, showed an evident change following RFR treatment [42]. 

Different outcomes emerged from most of the studies conducted in Wistar rats, for example when rats of the same strain were exposed to RFR emitted by an active cell phone at the GSM frequencies of 0.9 and 1.8 GHz for 1 h a day for 1 month, in comparison to a group of animals exposed for the same period to a no battery cell phone. Total sperm count was not affected, but sperm motility of experimental rats was significantly reduced by the exposure. The average percent of motile sperm reduced of about 40%, being 72.0% ± 8.7% in control rats and 43.1% ± 10.0% in RFR-exposed rats. Moreover, epididymis and testis of RFR exposed animals showed a marked increase in lipid peroxidation and a significant decrease in GSH content [43].

Kesari et al. (2010) exposed adult rats for 2 h/day up to 5 weeks to 900 MHz RFR with a level of SAR estimated to be 0.9 W/kg. Treated animals showed a significantly decreased total sperm count and level of protein kinase C (PKC), as well as an increase in apoptosis of spermatozoa. The enzyme PKC is commonly located in head, neck, and tail of human sperm, and plays an important role in the acrosomal reaction and sperm motility. The authors associated the reduction in PKC activity to the RFR-dependent possible overproduction of ROS in the sperm of exposed animals [44].

To investigate the hypothesis of an increased production of free radicals and other effects on fertility, the same researchers exposed the same strain of male rats to the same type and duration of RFR exposure. They found the antioxidant enzymes glutathione peroxidase and superoxide dismutase significantly lowered, and the catalase and malondialdehyde significantly increased in the exposed group, compared to the unexposed. Moreover, testicular sperm of exposed rats showed an increased content in micronuclei and a significantly changed cell cycle of G(0)–G(1) and G(2)/M. Generation of free radicals (ROS) was significantly increased in sperm [45].

In the same year, Meo and collaborators exposed male Wistar albino rats to GSM cell phone RFR for half an hour a day or for 1 h a day, for a 3 months period in total. In comparison to control rats, 18.75% of longer exposed rats showed the arrest of sperm maturation and hypospermatogenesis. The exposure to cell phone RFR for half an hour a day did not result in any abnormal findings in the animals [46]. 

In a study of 2012, Kesari and Behari exposed Wistar rats to cell phone RFR for 2 h a day for 45 days. Exposed rats showed a significant decrease in testosterone levels and an increased activity of caspase-3 protein, compared to controls. Transmission Electron Microscope observations also revealed sperm head and midpiece of sperm mitochondrial sheath distortions. Furthermore, this study revealed a reduction in litter size and weight of the progeny deriving from RFR-exposed male rats mated with unexposed females, compared to controls. The authors attributed these observations to ROS overproduction in rats exposed to cell phone RFR [47].

A long-term study analyzed the effects of exposure to cell phone emitting at 900 MHz on the reproductive organs of male rats. Specific levels of SAR for testis and prostate ranged from 0.0373 to 0.0623 W/kg. Exposed rats underwent RFR 3 h daily for one year. Once the experiment ended, the authors claimed that, under the condition used, RFR alter some reproductive parameters. In particular, the morphologically abnormal spermatozoa rates of treated rats were found significantly higher, in comparison to control rats. Moreover, at the histological examination of the seminiferous tubules, the Johnsen testicular biopsy score and the tunica albuginea thickness were found significantly decreased in the exposed animals [48].

In the same year, a study by Meena et al. aimed to evaluate the protective effect of the well-known antioxidant melatonin (MEL) in male Wistar rats, demonstrated that the prolonged RFR exposure could generate oxidative stress-mediated damage of the testes. The animals were divided into four groups: Controls (sham exposure); 2 mg/kg MEL treated; 2.45 GHz RFR exposed; and co-treated with RFR + MEL. Exposure was of 2 h a day for 45 days, and SAR was estimated at 0.14 W/Kg. Experimental observations showed that RFR biochemically induced oxidative damage by significantly decreasing testicular LDH levels and by increasing testicular MDA and ROS levels. Furthermore, RFR significantly affected the sperm count, the levels of testosterone, the fragmentation of DNA in testicular cells, the content of xanthine oxidase and carbonylated proteins [49].

Exposure effects on testes were also evaluated in another long-term study, in which rats underwent Wi-Fi-emitted 2.4 GHz RFR for 24 h a day during 12 months. Among the different parameters analyzed, the weight of the seminal vesicles and epididymis, the diameter of the seminiferous tubules and the thickness of tunica albuginea were found significantly lowered, compared to controls, while head defects and mitochondrial distribution alterations in the mid-piece of sperms significantly increased in the exposure group [50].

Adult male Wistar-Albino rats were long-term treated with 2.4 GHz RFR reproducing Wi-Fi exposure to evaluate the potential DNA damage on a series of different tissues. The detection of possible DNA damage was realized through the method of the single cell gel electrophoresis assay (comet). In exposed rats, the % tail DNA values of the liver, brain, kidney, and skin tissues were higher than that of the controls, but the increase resulted statistically significant only in testes tissue [51].

In a study conducted in 2018, Narayanan et al. investigated the possible adverse effects on blood biochemical and reproductive parameters of adolescent male albino Wistar rats exposed to 1h/day 900 MHz RFR from a mobile phone for 28 days. The treatment caused a slight reduction in sperm motility and a statistically significant increase in abnormal sperm percentage, in comparison to unexposed rats. Moreover, the testes of 900 MHz-exposed animals showed a loss of germ cells, in particular, spermatids and spermatocytes. Furthermore, the activity of testes caspase-3 was slightly increased, and MDA concentration was found increased in exposed rats [52].

The most recent study performed in Wistar rats for evaluating the effects of RFR on the male reproductive system was that of Gautam et al. in 2019. Rats underwent cell phone RFR exposure from 3G technology for 2 h/day for 45 days in specially designed exposure structures. A spermatogenic cells decrease was detected through histopathological examination, as well as sperm membrane and sperm tail morphology alterations. Among the various biochemical and physiological parameters analyzed, significant increases in lipid peroxidation and ROS levels with concomitant sperm count decrease and alteration in the mitochondrial activity of spermatozoa were observed [53].

The studies discussed in this section are summarized in Table 4.

#### 3.1.2. Mice

In the first study reported on mice, the animals were subjected to 0.09 W/kg RFR exposure, at 900 MHz. Polycarbonate cages were accommodated within an ad hoc built waveguide where mice were irradiated for 12 h/day for a week. The mitochondrial genome and the nuclear β-globin locus resulted significantly damaged when the DNA integrity was detailed analyzed through qPCR. Apart from this genotoxic effect in epididymal spermatozoa, this study did not document any other detrimental effect by RFR exposure on the development of male germ cell [54]. Nonetheless, it should be pointed out that in this study it was used a SAR value about 10 times lower than that used in the 2010 study by Kesari et al. conducted in Wistar rats [44]. The contrasting outcomes from these two studies may be partly explained by both the different experimental exposure conditions, and the different strain used (mice are much smaller than rats).

A 2010 study by Otitoloju et al. exposed male mice at residential quarters and a workplace complex to RFR from radio base antenna at 900 to 1800 MHz for six months, and compared to unexposed animals observed a statistically significant increase (39.78 and 46.03%, versus 2%, respectively) in the occurrence of sperm head defects. Such abnormalities were found to be dose-dependent, and mainly consisted of pin-head, banana-shaped and knobbed hook sperm head [55].

The study conducted by Pandey et al. in 2017 using Swiss albino mice investigated the effects of RFR exposure on male germ cell transformation kinetics, and evaluated the possible recovery. Animals were exposed to 900 MHz RFR for 4 or 8 h a day for a total of 35 days. Some animals were sacrificed after those 35 days of exposure, while others were given the opportunity to recover from exposure for further 35 days. The damage index of germ cells and the sperm head abnormalities were significantly increased in exposed mice. Flow cytometric estimation of germ cell subtypes in mice testis revealed 2.5-fold increases in spermatogonial populations with significant decreases in spermatids. A reduction of almost three times in primary spermatocyte to spermatid turnover, and a fourfold reduction in spermatogonia to spermatid turnover were found, to indicate a spermatogenesis arrest in the premeiotic stage. As a consequence, post-meiotic germ cells markedly decreased at the histological observation of the testes, as well as the sperm count lowered in mice exposed to RFR. Furthermore, histological alterations, such as epithelium depletion, maturation arrest and loss of immature germ cells into the seminiferous tubule lumen were also observed. Nevertheless, all the observed effects showed varying degrees of recovery when animals underwent to a post-treatment recovery period [56]. 

In a very recent experimental study, the same authors investigated the impact of GSM RFR at 900 MHz on germ cells development during spermatogenesis of Swiss albino mice. Animals were divided into four groups, one of which underwent RFR exposure 3 h a day twice, for 35 days, another received the same exposure with 5 mg/kg bw/day MEL supplementation, a third group received only MEL, and the last one remained unexposed. As consistent with the previous experiment, RFR exposure caused extensive DNA damage in germ cells, arrest in pre-meiotic stages of spermatogenesis, eventually leading to sperm head defects and low sperm count. Moreover, excess free radical generation was revealed through biochemical assays, thus, leading to histological and morphological changes, respectively in testis and germ cells morphology. These effects were either diminished or absent in RFR-exposed animals supplemented with MEL [57]. This result confirms the findings by Meena et al. in Wistar rats [49].

The studies discussed in this section are summarized in Table 5.

#### 3.1.3. Other Models

Two studies were performed by the same research group using adult male rabbits to evaluate the effect of mobile phone-emitted RFR on weekly collected semen samples. The two lagomorph studies were conducted with the identical experimental design and protocol of exposure and differed only in the frequencies used, which were of 900 MHz [58] and 800 MHz [59]. The whole-body average SAR was 0.43 W/kg in both studies. Mobile phone in standby mode was positioned close to the genitalia of the animal for 8 h/day over 12 weeks in order to assess testicular function. Salama et al. evaluated fructose and citrate levels, sperm motility and viability, serum testosterone levels, histological sections from the prostatic complex, ampulla, and vesicular gland. Compared to the unexposed group, there was a significant decrease in both fructose levels and number of motile sperms in the exposed group at the 10th week of exposure. There were no changes in citrate levels. A significant reduction in the sperm concentration in the exposed group at week 8 and a drop in sperm motility at week 10, accompanied by a significant decrease in the diameter of seminiferous tubules were also observed. The studies discussed in this section are summarized in Table 6.

### 3.2. Female Reproductive System and Other Reproductive/Developmental Endpoints

#### 3.2.1. Rats

Lary et al. exposed pregnant SD rats to 100 MHz RFR at a SAR level of 0.4 W/kg, for 6 h and 40 min/day. The exposure period was from gestation day 6 to 11, for a total of 40 h. The SAR value used corresponded to the maximum permissible level as indicated by the 1982 American National Standards Institute (ANSI) standard for RFR exposure. Irradiated rats did not differ in the percentage number of malformed fetuses, implantations per litter, percentage of implantations dead or resorbed, fetal weight, fetal crown-rump length, or fetal sex ratio, compared to untreated (sham-exposed) rats. The results showed a lack of embryotoxic or teratogenic effect in rats at the maximum permissible RFR exposure level following the 1982 ANSI recommendations [60].

A 2009 study by Ogawa et al. investigated the effect of 90 min/day 1.95 GHz CDMA emission on SD rats embryogenesis, exposing the dams from gestation day 7 to 17. All the animals were killed at gestational day 20, and the fetuses were extracted by cesarean section. The authors compared treated and untreated rats for placental weights, number of live, dead or resorbed embryos, sex ratios, weights, or visceral, external, or skeletal abnormalities of live fetuses Neither differences in maternal body weight gain, nor adverse effects of RFR exposure on any considered embryotoxic and reproductive parameters analyzed were observed [61].

In a study of 2010, Takahashi et al. evaluated the effects of 2.14 GHz RFR reproducing the emission of radio base antennas of mobile telephony. Pregnant SD rats were long-term whole-body exposed at two different exposure levels and compared to untreated animals. The calculated average SAR at the high exposure level ranged from 0.066 to 0.093 W/kg for the dams, and from 0.068 to 0.146 W/kg for the fetuses and the F(1) progeny. At the low exposure level, the SARs were estimated to be around 43% of those calculated for the high level. Treated rats underwent 20 h/day RFR exposure during gestation and lactation period. Parameters evaluated in dams were gestational conditions, organ weights and growth, while the F(1) generation was evaluated at 10 weeks of age for growth, development, survival rates, hormonal status, physical and functional development, memory function and reproductive ability. The F(2) offspring were analyzed for possible effects of teratogenicity and embryotoxicity. Both the RFR exposed dams and F(1) progeny showed no treatment-related effect for any of the parameters evaluated. The same was observed for the F(2) generation. The RFR exposure conditions used in this study did not determine any adverse effect on rat pregnancy or development [62].

Among the “non-influential” studies conducted in Wistar rats, two were conducted by Poulletier de Gannes and collaborators. Animals were exposed over two generations to a 2450 MHz Wi-Fi emission signal during gestation and lactation periods. In 2012, these researchers evaluated the possible adverse effects on pregnant rats and their F(1) generation of a Wi-Fi signal prenatal exposure of 2 h a day, 6 days a week, for a total of 18 days. The whole-body SARs used corresponded to 0.08, 0.4, and 4 W/kg. No statistically significant effects were noted at each dose tested in both dams and their pups, respectively in terms of observed abnormalities and pre- and postnatal development signs of toxicity [63].

The following year, another similar study from the same authors was conducted in rats of the same strain for investigating the exposure effects to a Wi-Fi signal with the same frequency on the male and female reproductive system. Adolescent animals were exposed for 1 h a day and 6 days a week to RFR, for three weeks in the case of males and for two weeks in the case of females. Afterwards, the rats were mated, and the couples treated for three more weeks. The day before parturition, clinical signs, abnormalities and mortality were evaluated in the fetuses. Under the condition used, the exposure to the Wi-Fi RFR signal did not determine any detrimental effect on fertility and reproductive organs either in male or in female rats. Likewise, the fetuses did not show any macroscopic abnormality [64].

Aït-Aïssa assessed immunological biomarkers in the sera of young Wistar rats RFR-exposed in utero and postnatally. Pregnant rats were located in a reverberation chamber and exposed to a Wi-Fi signal at the frequency of 2.45 GHz for 2 h a day and 5 days a week. Dams were whole-body exposed at average SAR values of 0, 0.08, 0.4, and 4 W/kg from gestation day 6 to 21. Furthermore, three newborns per litter were exposed for an additional 35 days. Antibodies directed against 15 different antigens related to damage and/or pathological markers were analyzed using the biochemical assay ELISA performed on sera of the experimental rats. The humoral response of the newborns showed no changes among different groups, for all the SAR levels used and the biomarker types considered. Some data on gestational outcome following in utero exposure to Wi-Fi signals were also provided by the present study; in particular, mass evaluation of dams and pups and the number of pups per litter were monitored, as well as the genital tracts of young rats were analyzed for abnormalities by measuring anogenital distance. Under the conditions used, the author’s findings indicate the absence of adverse effects, due to Wi-Fi signal exposure on general conditions and delivery in of Wistar rats [65].

One of the first evidence of teratogenicity, due to RFR in SD rats was that showed by Lary et al. in the early 1980s using low, non-thermal exposure level, with results in contrast with other findings from the same authors [60]. For investigating possible embryotoxic effects, in this study, pregnant SD rats were irradiated with 27.12 MHz RFR on gestation day 9 at an approximately SAR value of 11 W/kg. Treatment determined a relatively quick rise in the temperature of rat colony. As the duration of the exposure increased, embryotoxic and teratogenic effects of the RFR-induced hyperthermia also increased. Both the temperature of the dams and the amount of time the temperature remains high in the dams were responsible for embryotoxic and teratogenic effects, due to treatment-related hyperthermia [66,67].

A series of experimental studies conducted by Nelson and his research group investigated in depth the combinatory developmental toxicity effects of RFR at 10MHz and the organic compound 2-methoxyethanol (2ME). For this purpose, SD rats were exposed to the two agents individually or concurrently, in comparison to sham-exposed animals. The authors used highly diverse doses and timing of exposure of pregnant rats; after the sacrifice of the animals on gestation day 20, external malformations were evaluated in the examined progeny. Results showed that the adverse effects produced by both the treatments administered alone were enhanced when the agents were co-administered. In addition to a significant, dose-related, increased frequency of malformations, the combination treatment enhanced the severity of malformations too. This extended project demonstrated that the co-administration of RFR and 2ME synergistically induced teratogenic effects, but only at hyperthermic levels of RFR [68,69,70,71].

The same research group administered with methanol SD rat dams concomitantly exposed to RFR, in order to evaluate whether the interactive effects observed for RFR and 2ME were limited to this agent, or if similar synergies could be observed with other chemicals. After the sacrifice of the animals on gestation day 20, external malformations were evaluated in the examined progeny. When RFR or methanol was administered alone, the authors observed a statistically significant increase in the number of resorbed fetuses. The same was not observed when the agents were co-administered to experimental rats [72]. Further studies in the field of developmental toxicology would be needed in order to clarify these contradictory results about the complex role of the different agents in interacting with RFR. 

To evaluate the impact of the prenatal exposure to 1800 MHz RFR on bone development, pregnant SD rats were exposed for 6, 12, and 24 h/daily for a total of 20 days. The rats were inspected at the end of the day 60 after birth, and compared to the progeny of untreated dams. The increase in prenatal RFR exposure time caused a significant decrease in the levels of cartilage at rest and a significantly increased number of apoptotic myocytes and chondrocytes. Importantly, Erkut et al. demonstrated that an exposure of 24 h per day to RFR impaired tibia, ulna and femur development. Furthermore, a calcineurin activity decrease was observed in both muscle and bone tissues. This study indicated that the development of muscle and bone tissues was negatively impaired by prenatal exposure to 1800 MHz RFR [73].

The study by Oral et al., in 2006, investigated the potential adverse effects of RFR on the endometrial tissue of Wistar rat, with particular reference to RFR-induced oxidative stress and apoptosis. Animals were exposed to 900 MHz cell phone RFR for 30 min a day, for one month in total. In their study, the authors evaluated lipid peroxidation trough MDA as a marker of endometrial impairment induced by oxidative stress, and Bax, Bcl-2, caspase-3, and caspase-8 to assess apoptosis immunohistochemically. Based on the findings from this study, the electromagnetic fields emitted from cell phones may cause oxidative stress and endometrial apoptosis [74].

The studies discussed in this section are summarized in Table 7.

#### 3.2.2. Mice

Two studies regarding the potential RFR impact on mating were conducted in mice. In 2009, a large study performed in both female and male C57BL mice living over four generations under constant (life-long, 24 h/day) exposure to three doses of UMTS RFR at 1966 MHz investigated histological, physiological, behavioral and also reproductive parameters. Adult mice underwent whole-body RFR exposure at the average SARs of 0.08, 0.4, and 1.3 W/kg (means calculated at mating time), and were compared to control (sham-exposed) animals. This study did not report any RFR-induced significant effect on both male and female reproductive system, particularly for what concerns the offspring development, the appearance of reproductive organs, and the fertility potential [75].

In the study by Zhu et al. (2015), mice were treated for 15 days with continuous 900 MHz RFR for 4 h/day at average SAR of 0.731 W/kg. Ended the treatment, all the exposed mice were individually mated with three mature virgin females. After 7 days, each male mouse was moved to a new cage for mating with three other female mice. This process was repeated for four consecutive weeks in total. At gestation day 18, all female mice were sacrificed for examining uterine content and evaluating the putative mating. All the observations conducted during the one month-long mating period showed no statistically significant differences in untreated female mice mated with treated male mice as regards total and live/dead uterine implants and percentage of pregnancies, in comparison to females mated with control male mice. As consistent with the previous study, this study showed no RFR mutagenic potential on male germ line [76].

In three different works, Finnie and collaborators explored the impact of cell phone RFR exposure on fetal mouse brain development, investigating different outcomes. The common design contemplated the use of a specifically built 900 MHz exposure system, through which pregnant mice received for 1 h/day a far-field, whole body exposure at a SAR level of 4 W/kg from gestation day 1 to 19. In the first experiment, the authors explored the effect of cell phone exposure on blood-brain barrier (BBB) permeability in the immature mouse brain. On the 19th day of gestation, just before delivery, heads were collected from fetuses. The integrity of BBB was evaluated by immunohistochemistry using endogenous albumin as a vascular tracer in the cerebral cortex, thalamus, hippocampus, midbrain, cerebellum, medulla and basal ganglia. No albumin extravasation was found in brains from control or exposed mice, indicating no increase in vascular permeability of the considered regions of the fetal brain [77].

To study the expression of immediate early gene c-fos as a marker of neural stress, the authors collected fetal heads on gestational day 19 as in the previous experiment. No statistically significant change in the expression of c-fos was immunohistochemically observed in the same brain regions evaluated in the previous experiment. The lack of difference in c-fos immunoreactivity between control and exposed brains indicated no stress response in the brain of the fetuses following cell phone RFR exposure during gestation period [78].

In their third study, the same authors collected from each animal three coronal sections of the brain, including various anatomical regions to investigate a possible stress response given by heat shock proteins (HSPs) induction. The immunostaining of HSP25, 32 and 70 revealed no stress response. Exposure of mouse fetal brains to cell phone RFR during the gestation period did not cause any stress response by immunohistochemically evaluating HSPs [79].

Magras et al. in 1997 studied the possible adverse effects of RFR exposure on the prenatal development of mice. Mice were located in sites of different power densities around an “antenna park” and were mated five times in a row, until 118 pups were generated. Thus, newborns were weighed, measured, and macro- and microscopically examined. Authors observed a progressive decrease in the number of generated newborns per dam, leading to irreversible infertility [80]. 

The evaluation of RFR-induced possible toxic effect on mouse ovaries was the aim of the 2009 study by Gul et al. using 21 days old mouse female pups. Pregnant mice underwent cell phone RFR by placing mobile devices under the cages for the entire pregnancy period. Cell phones in standby position were turned on to speech position for 15 min every 12 h. Female newborns were sacrificed on day 21 after delivery, and the right ovaries were collected for determining the number of follicles. Findings from this study indicated a significantly decreased number of follicles in exposed pups compared to controls, indicating a toxic intrauterine effect of RFR on pup ovaries [81].

The studies discussed in this section are summarized in Table 8.

## 4. Discussion

Some long-term studies published up to the 2011 RFR IARC evaluation failed to demonstrate the carcinogenicity of RFR in experimental animals. RFR exposure has not been considerably linked to an increased incidence of tumors at any site both in rats or mice exposed for 24 months at different RFR intensities [13,14,15,25], and in transgenic and tumor-prone mouse strains [29,30]. If these studies have contributed to an increase in knowledge about potential RFR toxicity, critical limitations in the design of the majority of these studies seriously limit the usefulness of the information for properly evaluating the carcinogenic potential of RFR. Among the limitations are very short daily exposure durations (less than two hours a day) in strongly restrained experimental animals or exposed to radiations levels too low for properly evaluating the carcinogenicity of RFR [14,15,25] or had one exposure level only [13]. Moreover, most of these long-term studies did not justify the selected dose(s), and was characterized by poor dosimetry which did not consider the growth of the animals. Those studies using tumor-susceptible and genetically altered animals were not focused on evaluating RFR carcinogenic potential, but rather on investigating the effects in the specific target sites in that particular model [29,30]. Furthermore, some of the studies made with tumor-prone laboratory animals did not expose the animals to RFR for sufficient time (< 1 year), and for this reason, were not included in this review [82,83,84]. Lastly, additional points of criticism are the limited number of organs assessed for histopathology and inadequate group sizes (<50 animals/sex/group).

As reported in the present review, more in vivo bioassays on RFR have been conducted since the 2011 IARC review, most of which adopted improved exposure systems and more accurate measures of RFR dosimetry. In particular, the two long-term experimental studies by NTP and RI were performed for evaluating the effects of everyday human exposure to RFR electromagnetic fields. NTP doses have been established to mimic the localized exposure on body tissues from a cell phone placed near the body, and, are therefore, particularly higher than those used by the RI, that is instead similar to those found in our living and working environment to mimic the full-body human exposure generated by mobile telephony base antennas. Despite the differences, recently, both studies reported a statistically significant increased incidence of the same type of very rare glial malignant tumors of heart (schwannoma) and brain (glioma). These tumors involve the same cells of the acoustic nerve vestibular neurinoma observed in humans in certain epidemiological studies. Another fact supporting this consistency is that the Schwann cells are glial cells of the peripheral nervous system whose role is to form myelin, and are analogous to oligodendrocytes of the central nervous system. The NTP defined as clear evidence the carcinogenic activity of GSM-modulated 900 MHz RFR of mobile phones, particularly based on this statistically significant increase in the development of malignant heart Schwannoma in male SD rats [22]. Nonetheless, it must be reported that both NTP and RI used simulated mobile telephony signals emitted by generators, rather than real-life signals from cell phones and mobile telephony base antennas, respectively, and this represents a limit shared with many other studies discussed within this review. In fact, these simulated signals employ fixed parameters and no variability, thus, resulting in very different from the corresponding real emissions that instead vary constantly and unpredictably. This makes real-life signals more bioactive, and living organisms seem to have much less defense against highly variable environmental stressors [85,86]. Therefore, the use of simulated signals might also lead to an underestimation of the potential harmful effects.

As indicated by animal studies focusing on female reproductive system outcomes, the main targets of the potential adverse effect, due to RFR exposure are endometrial tissue, ovarian follicle numbers, granulosa cells, quality of oocytes and embryos during pregnancy. However, to date studies conducted in mammals regarding both female reproductive system and other reproductive/developmental endpoints are highly diverse, very inconsistently conducted and, most of all, report different specific outcomes, making it difficult to come to a conclusion about the different specific subjects. 

On the other side, the evidence from studies on male reproductive system suggest that RFR exposure might negatively affect male fertility. The increased sperm cell death rate accompanied by reduced sperm quality and motility seems to be the more recurring effects, due to RFR exposure in SD rats [38,39,40]. Guo et al., based on their findings from the most recent study conducted in SD rats suggested that the RFR-induced impaired sperm quality in SD rats might be accounted for by the increased apoptosis of testicular cells and the disruption of the secreting function of Leydig cells. Based on the available literature on male Wistar rats, most of the studies show that RFR exposure causes the decrease of sperm motility and number, and the increase of oxidative stress [43,44,45,47,49,52,53]. Sperm head abnormalities accompanied by an altered mitochondrial distribution, are also often reported [47,48,50,52,53]. Since an appropriate distribution of mitochondria plays a key role in sperm motility, the alterations found by some studies may explain the reported reduced sperm motility [47,50]. Most of the available mouse studies are in agreement that the oxidative stress induced by RFR exposure can cause DNA damage in germ cells. This genotoxic effect can alter cell cycle progression, causing decreased sperm count and motility, and abnormalities of the sperm head in mice [54,55,56,57]. Interestingly the two recent works by Pandey proved the MEL inhibitory effect, as well as the reversibility of such harmful effects in case of suspension of RFR exposure. The same authors also hypothesized that exposure to RFR led to mitochondrial membrane depolarization in mice germ cells which in turn results in altered cellular redox homeostasis [56,57].

Seminiferous tubules, spermatozoa and Leydig cells are the main targets of this damage, and sperm count, motility and morphology represent the more frequently affected parameters. The abnormalities highlighted in many studies are likely related in a direct manner to the duration of mobile phone use and/or to the proximity to the RFR source. Several studies support the hypothesis that RFR exposure causes an increase in oxidative stress, leading to DNA and sperm membrane lipid damage which eventually cause the aforementioned effects. Therefore, it is essential to conduct mechanistic studies for elucidating the manner in which RFR impairs biological function, thus, providing a solid rational cause. Moreover, more studies are needed to supply stronger evidence that RFR emitted from mobile telephony base antennas and the use of the cell phone alter sperm and gonads functions given the many limitations characterizing the existing literature. Nevertheless, based on the in vivo animal studies conducted so far, it is likely that RFR could negatively impact sperm damaging male human fertility, especially when the mobile phone is kept in active mode in an area close to testicles. 

## 5. Conclusions

In conclusion, according to NTP, there is now clear evidence that RFR causes cancer in experimental animals. RFR re-evaluation has also been listed as a priority by IARC [87]. There is also stronger evidence that RFR exposure is responsible for causing alteration of various sperm parameters, thus, affecting male fertility. Although a clear quantification of the carcinogenic and reproductive risk is still lacking, these animal findings suggest that a precautionary approach should be promoted by regulatory and health agencies, especially for children and pregnant women. Caution should also be considered in the development and spread of the upcoming 5G technology, particularly in light of the proposed higher frequencies and intensities of the signal. Long-term animal studies are urgently necessary to verify the possible health effects of 5G technology.

## Figures and Tables

**Table 1:** Studies of carcinogenicity in rats exposed at least two years to radiofrequency radiation (RFR).

Strain, Species (Sex) Duration Reference	RFR Exposure Levelfrequencies, Intensities (Any Other Co-Exposure)	Exposure Time No. of Animals	Increased Tumor Incidence (Significance)
Fischer 344 rats (M, F) 24 months La Regina et al., (2003)	835.62 MHz FDMA, 847.74 MHz CDMA 1.3 ± 0.5 W/kg	4 h/day, 5 days/week 80/sex/group	Not any increased tumor incidence (NS)
Fischer 344 rats (M, F) 24 months Anderson et al., (2004)	1600 MHz iridium signal Prenatal brain SAR (fetuses): 0.16 W/kg Brain SAR: 0.16 W/kg, 1.6 W/kg	2 h/day, 5 days/week 90/sex/group	Not any increased tumor incidence (NS)
Fischer 344 rats (M, F) 24 months Smith et al., (2007)	900, 1800 MHz (GSM, CDS) 0.44, 1.33, and 4.0 W/kg	2 h/day, 5 days/week 65/sex/group	Not any increased tumor incidence (NS)
Sprague-Dawley rats (M) 25 months Chou et al., (1992)	2450 MHz pulse modulated 0.144–0.4 W/kg	21.5 h/day, 7 days/week 200/group	Total primary cancers malignant lymphoma thyroid cancer (*p* < 0.05)
Sprague-Dawley rats (M, F) Before birth trough 24 months Wyde et al., (2016)	900 MHz (GSM, CDMA) 1.5, 3, 5 W/kg	9 h/day, 7 days/week 105/sex/group	Male brain glioma and heart Schwannoma (*p* < 0.05)
Sprague-Dawley rats (M, F) Before birth trough spontaneous death Falcioni et al., (2018)	1800 MHz (GSM) 0.1 W/Kg, 0.03 W/Kg, 0.001 W/Kg	19 h/day, 7 days/week Groups I,II: 400/sex/group Groups III, IV: 200/sex/group	Male heart Schwannoma (*p* < 0.05) and female brain glioma (NS)

M, males; F, female; h, hour(s); NS, not significant.

**Table 2:** Studies of carcinogenicity in mice exposed at least two years to RFR.

Strain, Species, (Sex) Duration Reference	RFR Exposure Level: Frequencies Intensities (Any Other Co-Exposure)	Exposure time No. of Animals	Increased Tumor Incidence (Significance)
B6C3F1 mice (M, F) Tillmann et al., (2007)	902 MHz (GSM) 1747 MHz (DCS) 0.4, 1.3, 4.0 W/Kg	2 h/day, 5 days/week 50/sex/group	Not any increased tumor incidence (NS)
B6C3F1/N mice (M, F) Before birth trough 24 months Wyde et al., (2016)	1900 MHz (GSM, CDMA) 2.5, 5, and 10 W/Kg	9 h/day, 7 days/week 105/sex/group	Not any increased tumor incidence (NS)
B6C3F1 mice (F) 24 months Tillmann et al., (2010)	UMTS fields 48 W/m^2^ and 4.8 W/m^2^ + prenatal ENU treatment of 40 mg/kg/b.w.	23.5 h/day, 7 days/week 60/group	Female lung carcinoma and lung tumor rate in ENU-pretreated group (tumor promotion) (*p* < 0.05)
B6C3F1 mice (F) 24 months Lerchl et al., (2015)	UMTS fields 0.04, 0.4, and 2 W/kg + prenatal ENU treatment of 40 mg/kg/b.w.	23.5 h/day, 7 days/week 96/group	Female lymphoma, lung adenoma and carcinoma, liver carcinoma (tumor promotion) (*p* < 0.05)

M, males; F, female; h, hour(s); NS, not significant.

**Table 3:** Studies of carcinogenicity in other models exposed at least two years to RFR.

Strain, Species, (Sex) Duration Reference	RFR Exposure Level: Frequencies Intensities (Any Other Co-Exposure)	Exposure Time No. of Animals	Increased Tumor Incidence (Significance)
E mu-Pim1 mice (lymphoma-prone) (M, F) 24 months Utteridge et al. (2002)	898.4 MHz GSM 0.25, 1.0, 2.0, and 4.0 W/kg	1 h/day, 5 days/week 120/sex/group	Not any increased tumor incidence (NS)
E mu-Pim1 mice (lymphoma-prone) (M, F) 18 months Oberto et al. (2007)	900 MHz pulse modulated 0.5, 1.4, 4.0 W/kg	1 h/day, 7 days/week 50/sex/group	Not any increased tumor incidence (NS)
E mu-Pim1 mice (lymphoma-prone) (F) 18 months Repacholi et al. (1997)	900 MHz GSM 0.008–4.2 W/kg, averaging 0.13–1.4 W/kg	1 h/day, 7 days/week 100/group	ẞ-cell lymphoma (*p* < 0.01)
C3H/HeA (breast cancer-prone) and Balb/c mice 12 months (M, F) Szmigielski et al. (1982)	2450 MHz 50, 150 W/m^2^ Balb/c mice also treated with 3, 4-benzopyrene (BP)	2 h/day, 6 days/week NR	Acceleration of breast tumor developed in C_3_H/HeA mice Acceleration of BP-induced skin cancer in Balb/c mice (*p* < 0.05)

M, males; F, female; h, hour(s); NS, not significant; NR, not reported.

**Table 4:** Male reproductive studies in rats exposed to RFR.

Strain, Species Reference	RFR Exposure Level Frequencies, Intensities (Any Other Co-Exposure)	Exposure Time No. of Animals	Endpoint(s) Impacted by RFR (Significance)
Sprague-Dawley rats Dasdag et al., (2003)	890–915 MHz (GSM) 0.52 W/kg	20 min/day, 7 days/week, 1 month 8/group	Not any statistically significant alteration (NS)
Sprague-Dawley rats Lee et al., (2010)	848.5 MHz 2.0 W/kg (CDMA)	90 min/day, 5 days/week, 12 weeks 20/group	Not any statistically significant alteration (NS)
Sprague-Dawley rats Lee et al., (2012)	848.5 MHz (CDMA), 1950 MHz (WCDMA) 4.0 W/kg	45 min/day, 5 days/week, 12 weeks 20/group (cage control group: 5)	Not any statistically significant alteration (NS)
Sprague-Dawley rats Imai et al., (2011)	1950 MHz (CDMA) 0.4 W/kg, 0.08 W/kg	5 h/day, 7 days/week, 5 weeks 24/group	Not any statistically significant alteration (NS)
Wistar rats Ribeiro et al., (2007)	1.835–1.850 GHz (GSM) 1.4 mW/cm^2^, 0.04 mW/cm^2^	1 h/day, 7 days/week, 11 weeks 8/group	Not any statistically significant alteration (NS)
Wistar rats Trošić et al., (2013)	915 MHz 0.6 W/kg	1 h/day, 7 days/week, 2 weeks 9/group	Not any statistically significant alteration (NS)
Sprague-Dawley rats Yan et al., (2007)	1900 MHz (CDMA) 1.80 W/kg (AMPS), 1.18 W/kg (PCS), 0.9 W/kg (CELL)	6 h/day, 7 days/week, 18 weeks 8/group	Decreased sperm motility (*p* < 0.05), abnormal clumping of sperm cells
Sprague-Dawley rats Qin et al., (2014)	1800 MHz 0.0405 W/kg	2 h/day, 7 days/week, 32 days 6/group	Decreased daily sperm production and motility, disruption of circadian rhythms, decreased testosterone levels, γ-GT and ACP activities, altered mRNA expression of P450 and StAR (*p* < 0.05)
Sprague-Dawley rats Guo et al., (2019)	220 MHz (pulsed modulated) 0.030 (whole body), 0.014 W/kg (testis)	1 h/day, 7days/week, 30 days 20/group	Decreased Leydig and Sertoli cells secreting factor levels, morphological alterations of the testis, increased levels of cleaved caspase 3, caspase 3, BAX/BCL2 ratio in the testis (*p* < 0.05)
Wistar rats Mailankot et al., (2009)	900, 1800 GHz (GSM) Intensities: NR	1 h/day, 7 days/week, 4 weeks 6/group	Decreased sperm motility, increase in lipid peroxidation and low GSH content in the testis and epididymis (*p* < 0.001)
Wistar rats Kesari et al., (2010)	900 MHz (GSM) 0.9 W/kg	2 h/day, 7 days/week, 5 weeks 6/group	Decreased level of PKC, total sperm count, and increased sperm cells apoptosis (*p* < 0.05)
Wistar rats Kesari et al., (2011)	900 MHz (pulse GSM) 0.9 W/kg	2 h/day, 7 days/week, 5 weeks 6/group	Decreased H1, GPx and SOD activities, and increased CAT activity, increased MDA, increased micronuclei and ROS, change in cell cycle of G(0)–G(1) and G(2)/M in sperm cells (*p* < 0.01)
Wistar rats Meo et al., (2011)	900, 1800 MHz (GSM) Intensities: NR	30 min/day, 60 min/day, 7 days/week, 12 weeks 16/group (control group: 8)	Hypospermatogenesis and maturation arrest in the testis (Significance: NR)
Wistar rats Kesari and Behari (2012)	900 MHz (GSM) 0.9 W/kg	2 h/day, 7 days/week, 45 days 6/group	Decreased testosterone level, increase in caspase-3 activity (*p* < 0.05), distortions in sperm head and mid piece of sperm mitochondrial sheath, reduction in litter size and weight of the progeny obtained from exposed males mated with unexposed females (*p* < 0.05)
Wistar rats Tas et al., (2014)	900 MHz (GSM) 0.0623 W/kg, 0.0445 W/kg, 0.0373 W/kg,	3 h/day, 7 days/week, 12 months 7/group	Decreased tunica albuginea thickness and the Johnsen testicular biopsy score (*p* < 0.05, *p* < 0.0001), increased morphologically abnormal spermatozoa rates (*p* < 0.05)
Wistar rats Meena et al., (2014)	2450 MHz 0.14 W/Kg (Melatonin 2 mg/kg bw/day)	2 h/day, 7 days/week, 45 days 6/group	Increased XO, DNA fragmentation and protein carbonyl content, decreased sperm count and testosterone level in testicular cells (*p* < 0.05)
Wistar rats Dasdag et al., (2015)	2400 MHz (from Wi-Fi system) 2420 μW/kg	24 h/day, 7 days/week, 12 months 8/group	Increased sperm head defects (*p* < 0.05), decreased weight of the epididymis and seminal vesicles, seminiferous tubules diameter and tunica albuginea thickness (*p* < 0.01, *p* < 0.001, *p* < 0.0001)
Wistar rats Akdag et al., (2016)	2400 MHz (from Wi-Fi system) 2420 μW/kg	24 h/day, 7 days/week, 12 months 8/group	Increased DNA damage (as percentage tail DNA value by Comet assay) in the testes
Wistar rats Narayanan et al., (2018)	900 MHz (GSM) 146.60 µW/cm^2^	1 h/day, 7 days/week, 4 weeks 6/group	Increased MDA and caspase 3 levels, reduced sperm motility (NS), increased percentage of abnormal sperm (*p* < 0.05), loss of germ cells (spermatocytes and spermatids) in the testes
Wistar rats Gautam et al., (2019)	1915 MHz (UMTS) 0.26 W/kg	2 h/day, 7 days/week, 45 days 8/group	Decreased weight of the sperm count (NS), viability and HOS tail-coiled spermatozoa (*p* < 0.05), increased MDA and ROS levels (*p* < 0.05, *p* < 0.01), reduced mitochondrial activity (*p* < 0.01), morphological alterations in sperm tail and membrane

h, hour(s); NS, not significant; NR, not reported; ROS, reactive oxygen species; GSH, glutathione; PKC, protein kinase C; H1, histone kinase; CAT, catalase; GPx, glutathione peroxidase; SOD, superoxide dismutase; XO, xanthine oxidase; MDA, malondialdehyde; HOS test, Hypo-Osmotic Swelling test; StAR, steroidogenic acute regulatory protein; γ-GT, γ-glutamyltransferase; ACP, acid phosphatase; T, testosterone; BAX, bcl-2-like protein 4; BCL2, B-cell lymphoma 2.

**Table 5:** Male reproductive studies in mice exposed to RFR.

Strain, Species Reference	RFR Exposure level Frequencies, Intensities (Any Other Co-exposure)	Exposure Time No. of Animals	Endpoint(s) Impacted by RFR (Significance)
CD1 Swiss mice Aitken et al. (2005)	900 MHz (GSM) 0.09 W/kg	12 h/day, 7 days/week, 1 week5/group	Damage in the mitochondrial genome and in the nuclear β-globin locus by DNA integrity analysis using qPCR
Albino mice, *mus musculus* Otitoloju et al. (2010)	900 to 1800 MHz (GSM) NR	24 h/day, 7 days/week, 6 months5/group	Increased sperm head abnormalities (knobbed hook, pin-head and banana-shaped sperm head) (*p* < 0.05)
Swiss albino mice, Pandey et al. (2017)	900 MHz (GSM) 0.0054 – 0.0516 W/kg	4 or 8 h/day, 7 days/week, 35 days15/group	Increased damage index in germ cells, sperm head defects, decreased sperm count, arrest in pre-meiotic stage of spermatogenesis, loss of immature germ cells into the seminiferous tubule lumen, epithelium depletion and maturation arrest (*p* < 0.05)
Swiss albino mice, Pandey et al. (2018)	900 MHz (GSM) (Melatonin 5 mg/kg bw/day) 0.0054 – 0.0516 W/kg	6 h/day, 7 days/week, 35 days 15/group	Decreased sperm count, sperm head abnormalities, extensive DNA damage in germ cells, arrest in pre-meiotic stages of spermatogenesis, excess free radical generation resulting in histological and morphological changes in testis and germ cells morphology (*p* < 0.05)

h, hour(s); NS, not significant; NR, not reported.

**Table 6:** Male reproductive studies in other models exposed to RFR.

Strain, Species Reference	RFR Exposure Level Frequencies, Intensities (Any Other Co-Exposure)	Exposure Time No. of Animals	Endpoint(s) Impacted by RFR (Significance)
Rabbits Salama et al. (2009)	900 MHz (mobile phone in standby mode) 0.43 W/kg	8 h/day, 7 days/ week, 12 weeks 10/sex/group	Decreased fructose levels in sperm and sperm motility (*p* < 0.05)
Rabbits Salama et al. (2010)	800 MHz (mobile phone in standby mode) 0.43 W/kg	8 h/day, 7 days/ week, 12 weeks 11/sex/group	Decreased sperm concentration and motility, decrease in the diameter of seminiferous tubules (*p* < 0.05)

h, hour(s); NS, not significant; NR, not reported.

**Table 7:** Female reproductive studies and other reproductive/developmental endpoints in rats exposed to RFR.

Strain, Species (Sex) Duration Reference	RFR Exposure Level Frequencies, Intensities (Any Other Co-Exposure)	Exposure Time No. of Animals	Evaluated Endpoint(s)	Endpoint(s) Impacted by RFR (Significance)
Sprague-Dawley rats (F) Lary et al., (1983)	100 MHz 0.4 W/kg	6 h 40 min/day, 6 days 3-10/group	Viable litter size/live birth index, neonatal growth, neonatal survival indices, prenatal mortality	Not any statistically significant alteration (NS)
Sprague-Dawley rats (F) Ogawa et al., (2009)	1950 MHz CDMA 0.4 W/kg	90 min/day, 7 days/week, 10 days 20/group	Landmarks of sexual maturity, viable litter size/live birth index, neonatal growth, neonatal survival indices, sex ratio in progeny, physiologic endpoints revealing unique toxicities of pregnancy and lactation	Not any statistically significant alteration (NS)
Sprague-Dawley rats (M, F) Takahashi et al., (2010)	2140 MHz (CDMA) Dams:0.066–0.093 W/kg Pups: 0.068–0.146 W/kg	20 h/day, 7 days/week, Gestation (3 weeks) + lactation (3 weeks) 4/group	Landmarks of sexual maturity, viable litter size/live birth index, neonatal growth, neonatal survival indices, sex ratio in progeny, physiologic endpoints revealing unique toxicities of pregnancy and lactation	Not any statistically significant alteration (NS)
Wistar rats (F) Poulletier de Gannes et al., (2012)	2450 MHz (CDMA Wi-Fi signal) 0.08, 0.4, 4 W/kg	2 h/day, 6 days/week, 18 days Prenatal study: 5 dams/group, and their pups Postnatal study: 15 dams/group, and their pups	Prenatal study: Number of live and dead fetuses per uterine horn, number and location in each uterine horn of early and late resorption sites, distribution of implantation sites on each uterine horn. Postnatal study: Landmarks of sexual maturity, viable litter size/live birth index, neonatal growth, neonatal survival indices, sex ratio in progeny, physiologic endpoints revealing unique toxicities of pregnancy and lactation	Not any statistically significant alteration (NS)
Wistar rats (M, F) Poulletier de Gannes et al., (2013)	2450 MHz (Wi-Fi signal) 0.08, 4 W/kg	1 h/day, 6 days/week, 5 weeks F 6 weeks M 12/group	Number of live and dead fetuses per uterine horn, number and location in each uterine horn of early and late resorption sites, distribution of implantation sites on each uterine horn	Not any statistically significant alteration (NS)
Wistar rats (F) Aït-Aïssa et al., (2012)	2450 MHz (Wi-Fi signal) 0.08, 0.4, 4 W/kg	2 h/day, 5 days/week, Dams: 15 days Pups: 5 weeks 12/ group	Landmarks of sexual maturity, viable litter size/live birth index, neonatal growth, neonatal survival indices, sex ratio in progeny, physiologic endpoints revealing unique toxicities of pregnancy and lactation	Not any statistically significant alteration (NS)
Sprague-Dawley rats (F) Lary et al., (1982, 1983)	27.12 MHz 11 W/Kg	30 min, Una tantum at gestation days 1, 3, 5, 7, 9, 11, 13, or 15, 30 min NR	Viable litter size/live birth index, neonatal growth, neonatal survival indices, prenatal mortality	Increased incidence of pre-implantation and post-implantation fetal malformations (*p* < 0.05), reduced fetal weight and crownrump length, increased incidence of dead or resorbed fetuses (*p* < 0.05)
Sprague-Dawley rats (F) Nelson et al., (1991, 1994, 1997, 1997)	10 MHz (2-methoxyethanol at 20, 40, 60, 75, 80, 100, 120, 125, 140 or 150 mg/kg) 0.8-6.6 W/Kg	10, 20, 30 min 10–27/group	Viable litter size/live birth index, neonatal survival indices, prenatal mortality	Synergism between RFR and 2ME administration in the induction of teratogenic effects: Increased incidence of external malformation of fetuses (*p* < 0.05)
Sprague-Dawley rats (F) Nelson et al., (2001)	10 MHz (Methanol 2, 3 g/kg) 0.8–6.6 W/Kg	60 min 10/group	Viable litter size/live birth index, neonatal survival indices, prenatal mortality	Increased incidence of resorbed fetuses (*p* < 0.05). No synergistic effects.
Sprague-Dawley rats (F) Erkut et al., (2016)	1800 MHz NR (217 Hz, pulse width of 577 µsec, maximum power 2 W)	6, 12, 24 h/day, 7 days/week, 20 days 12/group, control group 4	Prenatal bone and muscle tissues development	Reduction of resting cartilage levels, increased in the number of apoptotic chondrocytes and myocytes (*p* < 0.05), reduction in calcineurin activities in bone and muscle tissues
Wistar rats (F) Oral et al., (2006)	900 MHz 0.016–4 W/kg (Vitamin E 50 mg/kg, vitamin C 20 mg/kg)	30 min/day, 7 days/week, 30 days 8/group	Oxidative stress-induced endometrial impairment and apoptosis	Increased endometrial tissue levels of MDA, decrease in immunolabeling of caspase-3, caspase-8 and Bax, and increase of Bcl-2 (*p* < 0.01)

h, hour(s); NS, not significant; NR, not reported.

**Table 8:** Female reproductive studies and other reproductive/developmental endpoints in mice exposed to RFR.

Strain, Species Duration Reference	RFR Exposure Level Frequencies, Intensities (Any Other Co-Exposure)	Exposure Time No. of Animals	Evaluated Endpoint(s)	Endpoint(s) Impacted by RFR (Significance)
C57BL mice (M, F) Sommer et al. (2009)	1966 MHz (UMTS) 0.08, 0.4, 1.3 W/kg	24 h/day, 7 days/week, Multi-generation Study 128 M and 256 F over four generations (1M and 2F per cage)	Viable litter size/live birth index, neonatal growth, neonatal survival indices, prenatal mortality, assessment of sperm quality, weight and morphology of reproductive organs, mating and fertility indices and reproductive outcome, landmarks of sexual maturity, sexual behavior.	Not any statistically significant alteration (NS)
ICR mice (M) Zhu et al., (2015)	900 MHz 0.731W/kg	4 h/day, 7 days/week, 15 days 10/group	Mating and fertility indices and reproductive outcome	Not any statistically significant alteration (NS)
BALB/c mice (F) Finnie et al. (a) (2006), Finnie et al. (b) (2006) Finnie et al., (c) (2009)	900 MHz 4 W/kg	1 h/day, 7 days/week, 19 days 10/group	(a) Blood-brain barrier permeability in the immature brain of fetal heads; (b) immediate early gene c-fos expression as a marker of neural stress; (c) stress response by induction of heat shock proteins	Not any statistically significant alteration (NS)
BALB/c mice (M, F) Magras et al., (1997)	80–900 MHz (different power densities around an “antenna park”)	24 h/day, 7 days/week, 6 months (multi-generation study) 6/sex/group over five generations (118 newborns analyzed)	Infertility for dams and males, lethality for embryos, teratogenicity or the reduction in deformity for fetuses	Decreased number of newborns per dam, ending in irreversible infertility (NR)
Swiss mice (F) Gul et al., (2009)	NR (mobile phone in standby position for 11 h and 45 min, and in call position for 15 min) NR	12 h/day, 7 days/week, 21 days 30/group	Oocyte quantification in F pups (measurement of volumes of the ovaries and count of number of follicles in every tenth section)	Decreased number of follicles in mice ovaries, decreased ovarian volume (*p* < 0.01)

h, hour(s); NS, not significant; NR, not reported.
